# A generic assay for the identification of splicing variants that induce nonsense-mediated decay in Pompe disease

**DOI:** 10.1038/s41431-020-00751-3

**Published:** 2020-11-09

**Authors:** Atze J. Bergsma, Stijn L. M. in ’t Groen, Fabio Catalano, Manjiro Yamanaka, Satoru Takahashi, Toshika Okumiya, Ans T. van der Ploeg, W. W. M. Pim Pijnappel

**Affiliations:** 1 Department of Clinical Genetics, Erasmus MC Medical Center, Rotterdam, Netherlands; 2 Department of Pediatrics, Erasmus MC Medical Center, Rotterdam, Netherlands; 3 Center for Lysosomal and Metabolic Diseases, Erasmus MC Medical Center, Rotterdam, Netherlands; 4 Department of Laboratory Medicine, Shinshu University Hospital, Nagano, Japan; 5 Department of Pediatrics, Asahikawa Medical University, Hokkaido, Japan; 6 Department of Biomedical Laboratory Sciences, Faculty of Life Sciences, Kumamoto University, Kumamoto, Japan

**Keywords:** RNA splicing, Disease genetics, Reverse transcription polymerase chain reaction

## Abstract

DNA variants affecting mRNA expression and processing in genetic diseases are often missed or poorly characterized. We previously reported a generic assay to identify variants that affect mRNA expression and splicing in Pompe disease, a monogenic disorder caused by deficiency of acid α-glucosidase (*GAA*). However, this assay could miss mRNA that is subjected to degradation. Here, we inhibited mRNA degradation using cycloheximide and performed unbiased splicing analysis of all *GAA* exons using exon flanking RT-PCR and exon internal RT-qPCR. In four patients that were suspected of harboring splicing variants but for which aberrant splicing could not be detected in normally growing cells, we detected a total of 10 novel splicing events in cells treated with cycloheximide. In addition, we found that sequences of *GAA* introns 6 and 12 were naturally included in a subset of transcripts from patients and healthy controls, indicating inefficient canonical splicing. Identification of aberrant splicing caused by the common Asian variant c.546G>T allowed the development of an antisense oligonucleotide that promoted canonical *GAA* pre-mRNA splicing and elevated GAA enzymatic activity. Our results indicate that this extended generic splicing assay allows the detection of aberrant splicing in cases of mRNA degradation to enable functional analysis of unknown splicing variants and the development of targeted treatment options.

## Introduction

Pompe disease (Glycogen storage disease type II, OMIM 232300) is an autosomal inherited recessive disorder caused by the partial or complete deficiency of the lysosomal enzyme acid α-glucosidase (GAA) [1]. GAA catalyzes the breakdown of lysosomal glycogen to glucose and its loss results in a progressive and ubiquitous accumulation of lysosomal glycogen, ultimately leading to cellular dysfunction. Clinical manifestations of the disease are predominantly present in skeletal and cardiac muscle, with patients commonly showing hypotonia, muscle weakness and in classic infantile patients also hypertrophic cardiomyopathy.

Clinical signs of Pompe disease are confirmed by determination of residual GAA enzymatic activity in primary fibroblasts, muscle biopsies and/or leukocytes. Histochemistry is routinely implemented for rapid assessment of glycogen content when muscle biopsies are available. Data from enzymology and histochemistry are supported by genomic DNA sequencing of the *GAA* gene to identify disease-associated variants, described in the Pompe variant database at http://www.pompevariantdatabase.nl [2].

The nature of the disease-associated variant in the *GAA* gene correlates to a certain extent with the severity by which Pompe disease manifests, and its identification is important to predict the severity of disease progression as well as for genetic counseling [3]. Once the disease-associated variants are identified, their effect on residual GAA enzymatic activity is predicted based on multiple aspects, which include comparison to the clinical outcome of patients carrying the same variant and/or using in silico prediction tools [2]. Furthermore, coding variants can be studied in vitro by introduction of the variant of interest into a *GAA* cDNA construct and testing in a transient transfection experiment. These assays provide valuable information on the functional effects of specific variants. However, they do not provide functional information on possible effects on transcription and splicing of the *GAA* mRNA. Previously, we reported a generic method that analyzes the functional effect of *GAA* variants on pre-mRNA splicing and mRNA expression in primary fibroblasts [4]. However, this assay has a fundamental restriction: analysis of variants that alter canonical splicing can be complicated due to possible effect of nonsense-mediated decay (NMD). On average two-thirds of the transcripts are predicted to be aberrantly spliced resulting in the generation of premature termination codons due to changes in the reading frame. NMD hampers the detection of aberrantly spliced mRNA and hence prevents identification of variants that alter canonical splicing. Inhibition of mRNA degradation using cycloheximide may allow for determination of alternatively spliced mRNA transcripts that could otherwise not be detected [5, 6].

Determination of aberrant splicing is not only relevant for diagnosis but also for the development of novel therapies [7]. In recent years, antisense oligonucleotides (AONs), synthetic oligonucleotides that can bind and alter mRNA expression and/or processing, have been developed that are able to restore or modify mRNA expression in several diseases [8]. AONs have been also exploited to shift the balance of aberrantly spliced *GAA* pre-mRNAs towards canonically spliced mRNAs for several different splicing variants [9], including the common c.-32-13T>G (IVS1) variant in Pompe disease [10–12].

Here we analyzed *GAA* mRNA for aberrant splicing events that could lead to activation of the NMD pathway. Eleven new splicing events were identified, of which only one could also be detected without cycloheximide treatment. Interestingly, three aberrant splicing events were observed also in healthy fibroblasts, indicating that these variants occur naturally. One major aberrant splicing event caused by the common c.546C>T variant could be rescued using AONs, providing a potential therapeutic strategy for patients carrying this variant.

## Materials, subjects and methods

### Patients and controls

Patients were diagnosed with Pompe disease based on phenotype, GAA enzymatic activity in leukocytes and fibroblasts, and genomic DNA sequence analysis. Healthy control primary fibroblasts from skin biopsies were obtained from random healthy individuals. Patients and controls gave informed consent.

### Nomenclature & database submissions

Annotations used follow the guidelines set by the Human Genome Variation Society [13]. Reference sequences used for the annotation of *GAA* cDNA variants are NM_000152.3 and LRG_673t1.1. Position c.1 represents the first nucleotide of the translation start codon ATG located in exon 2. All disease-associated variants described in this manuscript are present in the Leiden open variation database (LOVD) (http://lovd.nl/gaa) and the Pompe disease *GAA* variant database (http://www.pompevariantdatabase.nl). The effects at the DNA, RNA and protein level and the corresponding variant and individual IDs and links are given in Table 1.

**Table 1:** Patient information.

Patient	Onset	Diagnostic value of GAA enzymatic activity (nmol 4-MU/hr/mg protein)^a^	Effect of variant at DNA level	Effect of variant at RNA level	Effect of variant at protein level	Leiden open variation database individual ID	Leiden open variation database variant ID	Link Pompe disease *GAA* variant database
Control	–	122.4	–	–	–	–	–	–
1	Classic infantile	1.5	c.1464dup	r.1464dup	p.Asp489Argfs*17	00306730	GAA_000302	Link
			c.1927G>A	r.[1927g>a,1755_1928del,1889_1928del]	p.[Gly643Arg,Leu587_Ala644del,Glu630Gly*53]		GAA_000021	Link
2	Childhood	3.7	c.546G>T	r.[-32_546del,546g>u,546g>u; 546_547ins546+1_546+184]	p.[0,=,Ile183Valfs*67]	00306731	GAA_000446	Link
			c.1798C>T	r.(1798c>u)	p.(Arg600Cys)		GAA_000092	Link
3	Classic infantile	1.6	c.1726G>C	r.(1726g>c)	p.(Gly576Arg)	00306732	GAA_000314	Link
			c.2481+2T>C	r.[2331_2332ins2332-109_2332-1; 2462_2481del,2462_2481del,2332_2481del]	p.[Val778AlaSerTer,Tyr822Profs*55,Val778_Gln827del]		GAA_000703	Link
4	Childhood	9.1	c.-32-13T>G	r.[=,-32_546del,-32_486del]	p.[=,0]	00306733	GAA_000029	Link
			c.2331+2T>A	r.[2315_2331delins2332-109_2332-1,2315_2331del]	p.[Trp772Cysfs*40,Trp772Cysfs*18]		GAA_000336	Link

^a^Enzymatic activity was determined at the Diagnostic Department of the Department of Clinical Genetics at the Erasmus MC, Rotterdam, Netherlands according to a validated diagnostic protocol using an internal control. The diagnostically determined healthy control range is 45–180 nmol 4-MU/hr/mg protein and the patient range is from 0 to 20 nmol 4-MU/hr/mg protein.

### Cell culture and cycloheximide treatment

Primary fibroblasts were obtained from skin biopsy and cultured in DMEM high glucose (Lonza, Basel, Switzerland) with 10% FBS (Hyclone, Logan UT, United States) and Penicillin/Streptomycin/Glutamine in (Thermo Fisher Scientific, Waltham MA, United States) 5% CO_2_ at 37 °C. Cycloheximide treatment was carried out at a concentration of 100 µg/ml cycloheximide (Sigma-Aldrich, Saint Louis MO, United states) for 48 h. A detailed description for the utilization of these parameters is provided in Supplementary Fig. S1.

### cDNA expression analysis

cDNA expression analysis was performed as shown previously in Bergsma et al. [9]. In short, *GAA* cDNA was introduced in the pcDNA3.1 expression vector. Site directed mutagenesis was used to introduce the c.1927G>A variant. WT and c.1927G>A vectors were transfected in HEK293T cells and cultured for 24 h. Cells were lysed and GAA activity was measured with the 4-MU analysis as described below. Samples were normalized for Neomycin expression to compensate for different transfection efficiencies as previously described [9].

### Transfection of AONs

AONs with a phosphorodiamidate morpholino oligomer backbone were obtained from Gene-Tools, LLC (Supplementary Table S1). Primary fibroblasts were transfected with AONs at 20 µM using 4.5 µl Endo-Porter transfection reagent (1 mM peptide in DMSO, Gene-Tools, Philomath OR, United States) per ml of medium. Cells were harvested after 3 or 4 days of culturing for mRNA analysis and 5 days of culturing for protein activity measurements.

### mRNA analysis

RNA was harvested and purified using the RNAeasy minikit (Qiagen, Hilden, Germany) according to manufacturer’s protocol. cDNA synthesis was performed on 800 ng RNA using iScript (Bio-Rad Laboratories, Hercules CA, United States) in a 20 µl reaction according to manufacturer’s instructions. cDNA was diluted 5x before further analysis. Flanking exon RT-PCR, in which the primers anneal to the exons upstream and downstream of the exon of interest (see Supplementary Table S2 for primer list), was performed using FastStart Taq Polymerase (Roche, Basel, Switzerland). RT-PCR was performed as an endpoint PCR with 35 cycles of amplification. Expression levels of each exon were quantified using RT-qPCR. RT-qPCR was carried out using iTaq universal SYBR Green Supermix (Bio-Rad Laboratories). The average of three technical replicates was used for calculation. *ACTB* levels were used as an internal reference for RT-qPCR analysis. Data were shown as the relative percentage of the values in a healthy control. CFX Manager analysis software (Bio-Rad Laboratories) was used to analyze the data. All primers used are shown in Supplementary Tables S2 and S3. A full description of the RT-PCR and RT-qPCR protocols has been described previously in Bergsma et al. 2015 [4]. Sequence analysis was performed directly on PCR samples using BigDye Terminator v3.1 (Thermo Fisher Scientific). If products were present at very low levels, TOPO cloning was performed using the TOPO® TA Cloning Kit (Thermo Fisher Scientific).

### Protein activity assay

Cells were lysed in standard lysis buffer (50 mM Tris (pH 7.5), 100 mM NaCl, 50 mM NaF, 1% Tx-100, Protease inhibitor). Protein quantification was done using the BCA Protein Assay according to manufacturer’s instructions (Thermo Fisher Scientific). 4-methylumbelliferone (4-MU)-α-D-glucopyranoside was used as substrate to measure GAA enzymatic activity [14].

## Results

We analyzed disease-associated variants that were suspected of affecting pre-mRNA splicing in four patients with Pompe disease (Table 1). Importantly, these variants showed no aberrant splicing products using our previously described splicing assay [4]. This assay involves analysis of splicing using flanking exon RT-PCR analysis of all exons in primary fibroblasts. Abnormal splicing events are identified based on product sizes and sequence analysis of splice products. We hypothesized that putative aberrant splice products could not be detected due to mRNA decay as a consequence of the introduction of a frameshift and/or a premature stop codon. Therefore, we adapted the generic splicing assay and introduced cycloheximide treatment to inhibit NMD and detect splice products that were normally degraded and escaped detection.

### Aberrant splicing of *GAA* pre-mRNA in primary fibroblasts from healthy controls

In order to establish a benchmark for the analysis of splicing following inhibition of NMD, primary fibroblasts from a healthy control were analyzed before and after treatment with cycloheximide.

**Fig. 1:**
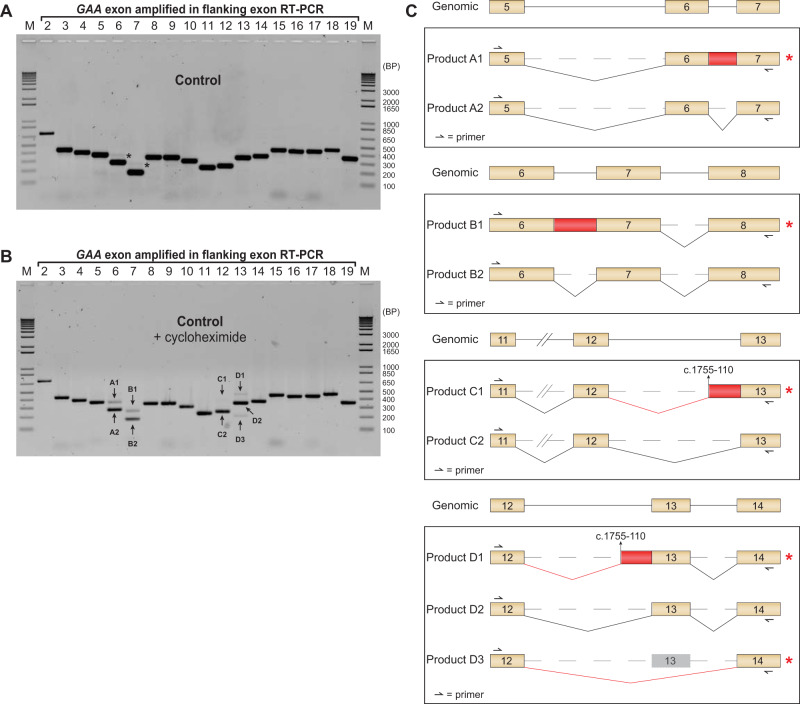
Identification of new splice products after inhibition of NMD in primary fibroblasts from a healthy control. **a** Flanking exon RT-PCR of *GAA* exons 2–19 in primary fibroblasts from a healthy control. Black asterisks indicate unidentified aberrant splice products. **b** Same as in **a**, but after treatment with cycloheximide. **c** Cartoons depicting products identified after sequencing of TOPO clones generated from the PCR samples of exon 6, 7, 12 and 13. Product labels refer to the numbers shown in **b**. Red lines indicate aberrant splicing events. Red asterisks indicate products that undergo NMD. Primer targets are indicated.

Flanking exon RT-PCR analysis of RNA from untreated control fibroblasts showed all the expected product sizes corresponding to canonical splicing of the *GAA* mRNA, as reported previously (Fig. 1a). Interestingly, faint products that were larger than the expected sizes were detected for *GAA* exons 6 and 7 (Fig. 1a, black asterisks). Treatment with cycloheximide showed an increased intensity of these products, and in addition revealed aberrant products around exons 12 and 13 (Fig. 1b). Products A1 and B1 were not unique to this cell line, since they were detected in five additional healthy control primary fibroblast cell lines tested (Supplementary Fig. S2A). Sequence analysis showed that *GAA* intron 6 was fully retained in products A1 and B1, causing a frameshift resulting in NMD (Fig. 1c, Supplementary Fig. S2B). Products A2 and B2 represent the canonical splice products of exons 6 and 7, respectively. Products C1 and D1 indicated abnormal splicing of exons 12 and 13. Sequencing analysis demonstrated that these products utilized a cryptic splice site in intron 12 at c.1755-110 that resulted in retention of the downstream intronic sequence up to exon 13, leading to the introduction of a stop codon and subsequent NMD (Fig. 1c and Supplementary Fig. S2C). Products C2 and D2 were normally spliced. In addition, a shorter product (D3) was detected in the flanking exon RT-PCR for exon 13, which resulted from a full skip of exon 13 (Fig. 1e and Supplementary Fig. S2F). Skipping of exon 13 led to NMD due to a shift in the reading frame.

Splice site strength prediction of all canonical splice sites of *GAA* using Alamut® software showed that the exon 6 donor splice site and the exon 7 acceptor splice site predicted an average splice site strength of 43 and 47%, respectively (Supplementary Fig. S2C). The average predicted values for all splice donor and acceptor sites in the *GAA* gene were 67 and 70%, respectively. This suggests that intron 6 retention may be caused by the weaker splice sites flanking intron 6. The prediction of the splice acceptor strength of exon 13 was close to the average (65%), while the predicted value of its donor site was high (93%) (Supplementary Fig. S2E and S2G), suggesting that aberrant splicing of exon 13 was not caused by low canonical splice site strength. The cryptic splice acceptor site at c.1755-110, which was utilized in products C1 and D1, had an average strength of 67%. Interestingly, four additional cryptic splice acceptor sites of similar strength were identified in between the utilized c.1755-110 site and the canonical splice site, however, these were not utilized for splicing in fibroblasts (Supplementary Fig. S2E).

These data indicate that in the absence of disease-associated variants, *GAA* pre-mRNA processing involves canonical splicing of all exons as well as aberrant splicing of exons 6, 7, 12 and 13. The aberrant splicing products are difficult to detect under standard cell culture conditions due to mRNA degradation, but their detection can be enhanced following cycloheximide treatment.

### Analysis of disease-associated variants in Patient 1

Patient 1 was diagnosed with classic infantile Pompe disease. Residual GAA enzymatic activity in primary fibroblasts was less than 2.0 nmol 4-MU/hr/mg protein. The common disease-associated c.1927G>A missense variant was identified on one allele [3]. cDNA expression analysis showed that this variant completely abrogates GAA enzymatic activity (Supplementary Fig. S3A). The disease-associated variant present on the second allele remained unknown. It should be noted that diagnosis for this patient was performed in 1982, leaving open the possibility of a missed 2^nd^ allele due to technical reasons.

**Fig. 2:**
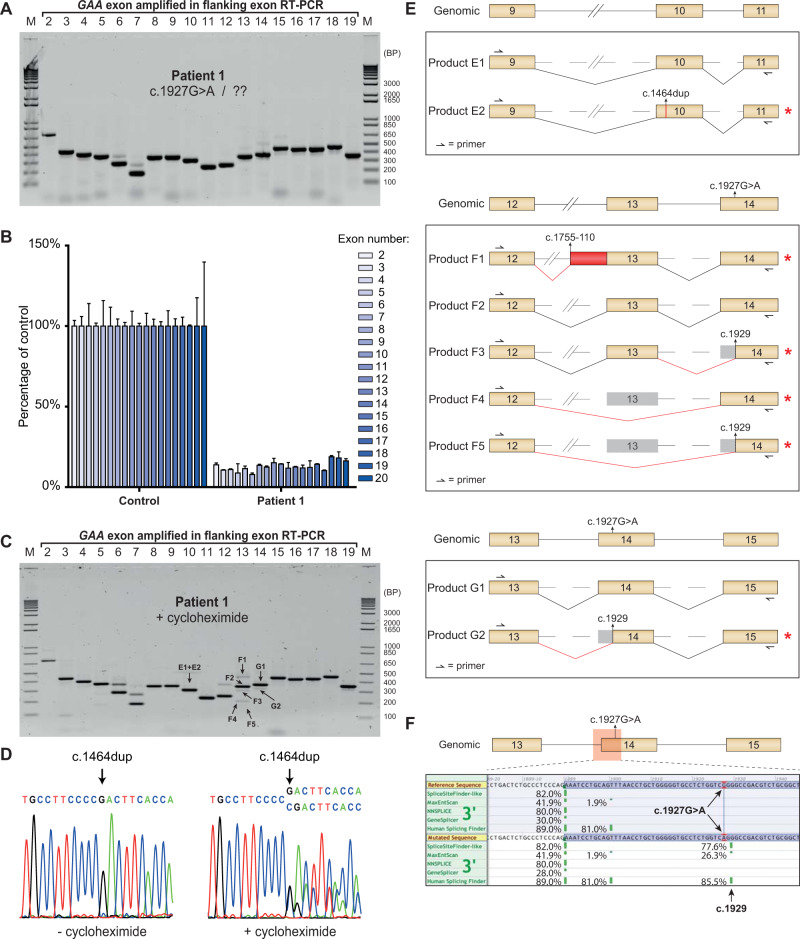
Analysis of aberrant splicing products in Patient 1. **a** Flanking exon RT-PCR of Patient 1 primary fibroblasts. **b** Exon-internal RT-qPCR for all coding exons of Patient 1 compared to healthy control. Data are shown as mean ± S.D. from three technical replicates. **c** Same as in **a**, but after cycloheximide treatment. **d** Sequencing results of flanking exon RT-PCR exon 10 sample with and without cycloheximide treatment. **e** Cartoons of products present in PCR samples for exons 10, 13 and 14. Product numbers refer to product highlighted in **c**. Red asterisks indicate products that undergo NMD. Red boxes indicate non-canonical sequences present in the *GAA* mRNA transcripts. Gray area’s highlight skipping of canonical *GAA* mRNA sequences. Primer targets are indicated. **f** Output of splice prediction performed in Alamut®. Five splice site prediction algorithms are shown for the area indicated in red, with or without the presence of the c.1927G>A variant. The location of the cryptic donor site utilized in products F3, F5 and G2 is highlighted.

Flanking exon RT-PCR analysis of primary fibroblasts derived from Patient 1 revealed no evident differences compared to the healthy control (Fig. 2a). Exon-internal RT-qPCR indicated an average expression level of all coding exons of 12% compared to the healthy control, which suggested that both alleles had reduced expression levels (Fig. 2b). Fibroblasts from Patient 1 were subjected to cycloheximide treatment to identify mRNA transcripts that might have underwent decay. Multiple aberrant products were identified by flanking exon RT-PCR and sequence analysis (Fig. 2c). Sanger sequence analysis of the RT-PCR products of exon 10 (Products E1 and E2) indicated a shift of the reading frame in half of the transcripts, corresponding with the insertion of one cytosine (c.1464dup, Fig. 2d and e). The c.1464dup variant therefore represented the disease-associated variant on the second allele and caused a frameshift resulting in a premature stop codon and subsequent mRNA decay. This is supported by the observation that in the absence of cycloheximide treatment, the only allele that was expressed was the c.1927G>A allele, while after cycloheximide treatment, expression of both the c.1927G>A and the c.1464dup allele could be detected (Fig. 2d).

Additional aberrant products were identified in the area surrounding exons 13 and 14, close to the c.1927G>A missense variant of the first allele. Sequencing analysis demonstrated that products F3, F5 and G2 utilized a cryptic splice acceptor site at position c.1929. All of these products underwent decay as a result of the introduction of a frameshift. Products F1 and F4 corresponded to the aberrant splice products D1 and D3, respectively, that were also detected in the healthy control, (Figs. 1c and 2e, Supplementary Fig. S3B). Products F2 and G1 were normally spliced.

In silico prediction was in agreement with these results and indicated that the c.1927G>A variant affected pre-mRNA splicing by generation of a cryptic splice acceptor at position c.1929 that competes with the canonical exon 14 splice acceptor site (Fig. 2f). This showed that the c.1927G>A missense variant caused aberrant splicing.

In conclusion, Patient 1 carried two disease-associated variants. Allele 1 harbored the known c.1927G>A missense variant. Although the missense variant by itself was very severe, it also led to aberrant splicing and mRNA decay due to the generation of a cryptic splice acceptor site at c.1929. Some leaky wild type splicing remained, resulting in a total residual mRNA expression of ~12% from this allele. The c.1464dup variant on the second allele that was identified here is a novel, very severe variant that causes a frameshift and mRNA decay.

### Analysis of the c.546G>T variant in Patient 2

Patient 2 was previously described by Takahashi et al. [15]. Analysis of primary fibroblasts from Patient 2 indicated a residual GAA enzymatic activity of 3.7 nmol 4-MU/hr/mg protein. Diagnostic sequence analysis identified two disease-associated variants. The first allele carried the c.546G>T synonymous variant that is located at the last base of *GAA* exon 2 and was known to cause aberrant splicing [16]. However, the functional consequences of this variant have not yet been fully elucidated. The second allele harbored the c.1798C>T missense variant. This variant is frequently observed in Japanese Pompe patients, and is described as very severe in the Pompe disease *GAA* variant database (http://www.pompevariantdatabase.nl) [17].

**Fig. 3:**
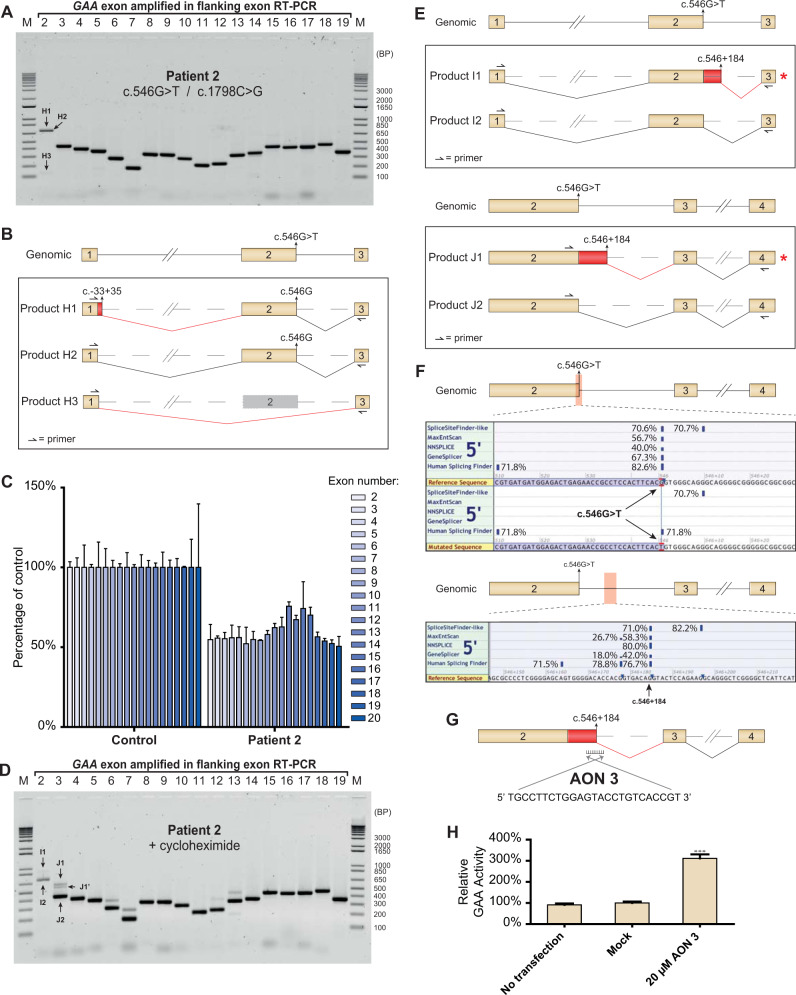
Analysis of aberrant splicing products in Patient 2. **a** Flanking exon RT-PCR of Patient 2 primary fibroblasts. **b** Cartoons of products identified to be present in PCR samples for exon 2. Product numbers refer to product highlighted in **a**. **c** exon-internal RT-qPCR for all coding exons of Patient 2 compared to healthy control. Data are shown as mean ± S.D. from three technical replicates. **d** Same as in **a**, but after cycloheximide treatment. Product J1’ indicates the same product as product J1. **e** Cartoons of products present in PCR samples for exon 3. Product numbers refer to product highlighted in **d**. Red asterisks indicate products undergoing NMD. Red boxes indicate non-canonical sequences present in the *GAA* mRNA transcripts. **f** Output of splice prediction performed in Alamut®. Upper panel shows five splice site prediction algorithms for the area indicated in red, with or without the presence of the c.546G>T variant. The lower panel shows predicted splice site strength surrounding the location of the cryptic donor site at c.546 + 184 utilized in products I1 and J1. **g** Cartoon depicting the target site and sequence of AON intron 2. **h** GAA enzymatic activity of Patient 2 fibroblasts after transfection with AON intron 2. Data are normalized for mock transfection and are shown as the mean ± SD of three biological replicates (***p = 0.001).

Flanking exon RT-PCR analysis on *GAA* mRNA was performed on primary fibroblasts from Patient 2. Products H1, H2, and H3 were identified for exon 2, of which H1 and H3 were only barely visible (Fig. 3a). Sequencing of these products led to the identification of three distinct splicing events. Product H2 corresponded to canonical splicing of exon 2. Product H1 represented the inclusion of 35 bases of intron 1 followed by normal splicing of exon 2. This concerns low abundant, normal splicing as reported previously by us and others [10, 18]. Product H3 was a full skip of exon 2 (Fig. 3b, Supplementary Fig. S4A). No other aberrant splice products were identified. Next, exon internal RT-qPCR was performed. *GAA* exons were expressed at an average level of 59.1% compared to the healthy control (Fig. 3c). To test if any abnormal splicing events were missed, primary fibroblasts from Patient 2 were treated with cycloheximide. Flanking exon RT-PCR identified the normally spliced products I2 and J2 for exons 2 and 3, respectively. Interestingly, the aberrant products H2 and H3 (Fig. 3a and b) could not be detected after treatment with cycloheximide. However, two additional products (I1 and J1) were visible, which indicated the presence of an additional aberrant splicing event (Fig. 3d). Sequence analysis of the aberrant products revealed that an aberrant transcript was generated that did not utilize the canonical splice donor site of exon 2, but instead utilized a cryptic splice donor site located at c.546+184 in intron 2 (Fig. 3e, Supplementary Fig. S4A). This mRNA transcript was likely degraded due to a frameshift caused by the retention of 184 bases of intron 2. Product J1’ indicated a product for which no sequence could be identified that corresponded with its size. Further investigation showed that this product represented a denatured form of product J1, that was potentially caused by rapid cooling of the PCR product (Supplementary Fig. S4B).

In silico analysis predicted that the canonical exon 2 splice donor site was poorly recognized in the presence of the c.546G>T variant (Fig. 3f), which indicated that the variant likely has an effect on splicing. In addition, the cryptic splice site at c.546+184 that was identified using the functional assay could also be predicted using Alamut® (Fig. 3f). A comparison of splice site strengths revealed that the canonical c.546G splice donor had the strongest predicted strength (Fig. 3f and Supplementary Table S4). The change of c.546G into c.546T resulted in a complete loss of predicted splice site strength in 4 out of 5 prediction algorithms, resulting in a predicted dominance of c.546 + 184 over the c.546T splice site.

We hypothesized that the cryptic splice donor site at c,546+184 could potentially be blocked using an AON to promote canonical exon 2 splicing. To test this hypothesis, five AONs that overlap the cryptic splice donor site at c.546+184 were designed and tested in primary fibroblasts from Patient 2 (Supplementary Fig. S4C). Enzymatic activity increased after blockage by AON 2 and AON 3 (Supplementary Fig. S4D). An additional experiment was performed in which only the most optimal AON (AON 3) was tested (Fig. 3g). This treatment resulted in a three-fold increase of GAA enzymatic activity (Fig. 3h). Furthermore, flanking exon RT-PCR of *GAA* exon 3 demonstrated that expression of the aberrant product was abolished following treatment of Patient 2 primary fibroblast cells with both AON 3 and cycloheximide, indicating that AON 3 indeed blocks utilization of the cryptic splice site at c.546+184 (Supplementary Fig. S4E).

In conclusion, the c.546G>T variant resulted in a reduced strength of the canonical *GAA* exon 2 splice donor site, resulting in skipping of exon 2, and to partial inclusion of intron 2 due to the utilization of a cryptic splice donor site at c.546+184. The identification of the utilized cryptic splice donor site allowed the design of an AON that was able to increase endogenous expression of functional *GAA* mRNA.

### Analysis of the c.2331+2T>A and c.2481+2T>C variants in Patients 3 and 4

Two additional patients were analyzed to assess the effect of the c.2331+2T>A and c.2481+2T>C variants on splicing. The c.2331+2T>A, that is located at the splice donor site of *GAA* exon 16, predominantly led to alternative splice donor utilization at c.2315 in the same exon. Similarly, the c.2481+2T>C variant, which is located at the splice donor site of *GAA* exon 17, resulted in utilization of a cryptic donor splice site located c.2462. In both cases, the utilization of the cryptic donor sites results in a frameshift, the generation of a premature stop codon and mRNA decay. A detailed description of the results is outlined in the Supplementary Text and Supplementary Figs. S5–S8.

## Discussion

The functional consequences of variants on pre-mRNA splicing often remain undetermined in current standard diagnostic analysis of monogenic disorders. Our study shows that inhibition of mRNA degradation and subsequent splicing analysis resulted in the identification of aberrant mRNA transcripts in four patients for which the conventional splicing assay (without inhibition of mRNA degradation) failed to detect aberrant splicing. The assay also revealed that aberrant splicing occurs in healthy controls that do not harbor disease-associated variants, indicating that not all mRNAs transcribed from the *GAA* gene produce functional protein. Moreover, we demonstrated that the results of the splicing assay combined with cycloheximide treatment can be used for the development of an antisense-based approach that can restore GAA enzyme activity in cells with the common Asian c.546G>T variant.

Nine percent of disease-associated variants have been described to affect splicing [19]. However, it has been estimated that as much as one-third of disease- causing variants could have an effect on splicing [20]. Functional splicing screening assays are often not a standard procedure in diagnostic labs. Variants that are classified as missense or nonsense variants could potentially (also) exert their pathogenic effect on pre-mRNA splicing, highlighting the need for better functional evaluation of disease-associated variants [21]. This also applies to variants that modulate splicing outcome but do not cause disease on their own, such as the silent c.510C>T *GAA* variant in Pompe disease that worsens splicing outcome of the IVS1 variant [22]. Aberrant splicing of *GAA* also occurs without the presence of disease-associated variants. Here we show that intron 6 retention takes place in all cell lines tested. Our previous findings suggest that this might occur due to the presence of weak splice sites [4]. Common single nucleotide variants could also have a modifying role in aberrant splicing of intron 6 and intron 12 [23]. Future work is required to delineate the cause of naturally occurring aberrant splicing in the *GAA* gene.

For Pompe disease, comprehensive in silico splice prediction analysis of *GAA* variants has previously been performed [2, 24]. We show that the splicing assay is able to detect aberrant splicing in an unbiased manner. An example is the *GAA* missense variant c.1927G>A present in Patient 1. The variant was first described in 1993 and has been classified as a very severe missense variant and is frequently identified in patients with Pompe disease [25]. We describe here that the pathogenic effect of this variant has two mechanisms: it alters the protein sequence, but also affects splicing of *GAA* exon 14. In this instance, the pathogenic effect of the missense variant led to complete abrogation of GAA enzymatic activity (Supplementary Fig. S3A). This rendered the pathogenic effect of aberrant splicing caused by this variant negligible. However, missense variants that affect splicing but allow some residual enzymatic activity could be targeted using splice switching AONs to partially restore enzymatic activity. An example is the c.1256A>T *GAA* variant [9]. This variant causes a change in protein sequence, but has little effect on enzymatic activity. Instead, c.1256A>T generates an exonic splice donor that causes aberrant pre-mRNA splicing. An AON was designed to restore aberrant splicing. Finally, there was partial overlap between the effects of the IVS1 and c.546G>T variants: both c.546G>T and IVS1 caused skipping of exon 2, but IVS1 caused in addition partial skipping of exon 2 and inclusion of a pseudoexon in intron 1 [11, 18, 26, 27]. The latter two events were not observed in c.546G>T cells, but instead these cells showed partial retention of intron 2. The likely cause for these differences is that IVS1 affects the splice acceptor of exon 2, while c.546G>T weakens the splice donor of exon 2. As a consequence, while both c.546G>T and IVS1 caused exon 2 skipping, AONs targeting distinct cryptic splice sites were required to correct these variants: AONs that blocked the cryptic splice donor and/or acceptor site of a pseudoexon in intron 1 in the case of IVS1 [10, 11], and AONs that blocked the cryptic splice donor site at c.546 + 184 in the case of c.546G>T (this study). In addition, AONs targeting exon 2 were found to be effective in restoring aberrant splicing caused by the IVS1 variant but not c.546G>T [12, 28]. These findings highlights the importance of functional splicing analysis for diagnostics and the design of treatment options.

Whole exome and whole genome sequencing are currently being implemented for diagnostic purposes at an increasing rate [29]. Although these techniques aid in identification of new disease-associated variants and genes, they do not provide functional data on variants affecting gene expression and/or pre-mRNA splicing. RNA-sequencing would give insight into both of these parameters. Implementation of this technique in routine diagnostics could potentially give a better understanding of functional consequences of disease-associated variants [30] and is currently being optimized for diagnostic implementation [31]. However, caution should be taken in interpretation of the results, since pre-mRNA splicing can be cell type specific [32]. For Pompe disease, in which muscle tissue is mainly effected, skeletal muscle cells would be the most optimal type of tissue to perform splicing analysis, but primary muscle tissue is difficult to obtain due to the high burden to the patients. In addition, primary human myoblasts have limited capacity for self-renewal in vitro, and go into senescence and fail to differentiation upon extended culturing. A solution could be the direct differentiation of patient-derived induced pluripotent stem cells (iPSCs) towards the muscle lineage. This has been utilized previously by us to compare aberrant splicing caused by the common c.-32-13T>G variant between primary fibroblasts and iPSC-derived muscle cells from the same patient [10]. No apparent differences in the splicing pattern were seen between the two cell types. In the most optimal scenario aberrant splicing should be examined in the relevant cell type, i.e., skeletal muscle in the case of Pompe disease, but at present the procedure to generate iPSC-derived skeletal muscle cells is expensive and time consuming.

The results obtained here can be important for the development of new variant-specific treatment strategies, such as splice-switching AONs. With the FDA approval of several antisense-based drugs, this treatment approach has received a substantial interest [33–35]. It represents a promising approach, for example by shifting the balance of aberrantly spliced pre-mRNAs toward canonical splicing and thereby restoring endogenous expression of the primary disease target. For Pompe disease several AONs have been developed, including AONs targeting rare variants [9] and AONs that prevent aberrant splicing caused by the IVS1 variant [10–12]. Furthermore, AONs that prevent aberrant splicing caused by the IVS1 variant can potentially be used to rescue aberrant splicing resulting from other variants present in *GAA* exon 2 [28]. Here we described the design of a novel AON that targets aberrant splice products generated due to the presence of the c.546G>T variant and enhances GAA enzyme activity in cells carrying this variant. The c.546G>T variant is frequent in Japanese patients with Pompe disease [36], and provides a target for a potential new treatment using AONs for these patients. The main barrier toward clinical development of AONs that target skeletal muscle is the limited uptake of systemic applied AONs in this tissue. Promising developments are ongoing to improve the chemistries of AONs for clinical development, which have so far resulted in approval of 10 AONs and numerous clinical trials [35]. We anticipate that this may accelerate clinical development of AONs for muscle disorders.

In conclusion, the extended splicing assay can be used to detect splicing events that otherwise remain undetected due to mRNA decay. A generic splicing assay combined with cycloheximide treatment should in principle also be feasible for other monogenic diseases besides Pompe disease to aid in the identification of variants that affect mRNA processing. Since splicing is amenable to modulation using AONs, the identification of novel splicing variants could be instrumental for the development of new therapies.

## Supplementary information

Supplementary data

## References

[1] van der Ploeg AT, Reuser AJ (2008). Pompe’s disease. Lancet.

[2] Nino MY, In ‘t Groen SLM, Bergsma AJ, van der Beek N, Kroos M, Hoogeveen-Westerveld M, et al. Extension of the Pompe mutation database by linking disease-associated variants to clinical severity. Hum Mutat. 2019;40:1954–67. 10.1002/humu.23854 PMC6851659 31254424

[3] Kroos M, Hoogeveen-Westerveld M, van der Ploeg A, Reuser AJ (2012). The genotype-phenotype correlation in Pompe disease. Am J Med Genet C Semin Med Genet.

[4] Bergsma AJ, Kroos M, Hoogeveen-Westerveld M, Halley D, van der Ploeg AT, Pijnappel WW (2015). Identification and characterization of aberrant GAA pre-mRNA splicing in pompe disease using a generic approach. Hum Mutat.

[5] Peccarelli M, Kebaara BW (2014). Regulation of natural mRNAs by the nonsense-mediated mRNA decay pathway. Eukaryot Cell.

[6] Schneider-Poetsch T, Ju J, Eyler DE, Dang Y, Bhat S, Merrick WC, et al. Inhibition of eukaryotic translation elongation by cycloheximide and lactimidomycin. Nat Chem Biol. 2010;6:209–17. 10.1038/nchembio.304 PMC2831214 20118940

[7] Bergsma AJ, van der Wal E, Broeders M, van der Ploeg AT, Pim, Pijnappel WWM (2018). Alternative splicing in genetic diseases: improved diagnosis and novel treatment options. Int Rev Cell Mol Biol.

[8] Levin AA (2019). Treating disease at the RNA level with oligonucleotides. N. Engl J Med.

[9] Bergsma AJ, In ‘t Groen SL, Verheijen FW, van der Ploeg AT, Pijnappel W (2016). From cryptic toward canonical pre-mRNA splicing in Pompe disease: a pipeline for the development of antisense oligonucleotides. Mol Ther Nucleic Acids.

[10] van der Wal E, Bergsma AJ, van Gestel TJM, In ‘t Groen SLM, Zaehres H, Arauzo-Bravo MJ (2017). GAA deficiency in Pompe disease is alleviated by exon inclusion in iPSC-derived skeletal muscle cells. Mol Ther Nucleic Acids.

[11] van der Wal E, Bergsma AJ, Pijnenburg JM, van der Ploeg AT, Pijnappel W (2017). Antisense oligonucleotides promote exon inclusion and correct the common c.-32-13T>G GAA splicing variant in Pompe disease. Mol Ther Nucleic Acids.

[12] Goina E, Peruzzo P, Bembi B, Dardis A, Buratti E. Glycogen reduction in myotubes of late-onset Pompe disease patients using antisense technology. Mol Ther. 2017;25:2117–28. 10.1016/j.ymthe.2017.05.019 PMC5589062 28629821

[13] den Dunnen JT, Dalgleish R, Maglott DR, Hart RK, Greenblatt MS, McGowan-Jordan J (2016). HGVS recommendations for the description of sequence variants: 2016 update. Hum Mutat.

[14] Kroos MA, Pomponio RJ, Hagemans ML, Keulemans JL, Phipps M, DeRiso M (2007). Broad spectrum of Pompe disease in patients with the same c.-32-13T->G haplotype. Neurology.

[15] Takahashi S, Tanaka R, Okano S, Okayama A, Suzuki N, Azuma H. Reduced efficacy of enzyme replacement therapy in a child with late-onset Pompe disease. Pediatrics Therapeutics. 2016;6:2.

[16] Maimaiti M, Takahashi S, Okajima K, Suzuki N, Ohinata J, Araki A (2009). Silent exonic mutation in the acid-alpha-glycosidase gene that causes glycogen storage disease type II by affecting mRNA splicing. J Hum Genet.

[17] Tsujino S, Huie M, Kanazawa N, Sugie H, Goto Y, Kawai M (2000). Frequent mutations in Japanese patients with acid maltase deficiency. Neuromuscul Disord.

[18] Dardis A, Zanin I, Zampieri S, Stuani C, Pianta A, Romanello M (2014). Functional characterization of the common c.-32-13T>G mutation of GAA gene: identification of potential therapeutic agents. Nucleic Acids Res.

[19] Stenson PD, Mort M, Ball EV, Evans K, Hayden M, Heywood S (2017). The Human Gene Mutation Database: towards a comprehensive repository of inherited mutation data for medical research, genetic diagnosis and next-generation sequencing studies. Hum Genet.

[20] Lim KH, Ferraris L, Filloux ME, Raphael BJ, Fairbrother WG (2011). Using positional distribution to identify splicing elements and predict pre-mRNA processing defects in human genes. Proc Natl Acad Sci USA.

[21] Soemedi R, Cygan KJ, Rhine CL, Wang J, Bulacan C, Yang J (2017). Pathogenic variants that alter protein code often disrupt splicing. Nat Genet.

[22] Bergsma AJ, In ‘t Groen SLM, van den Dorpel JJA, van den Hout H, van der Beek N, Schoser B (2019). A genetic modifier of symptom onset in Pompe disease. EBioMedicine.

[23] Lappalainen T, Scott AJ, Brandt M, Hall IM (2019). Genomic analysis in the age of human genome sequencing. Cell.

[24] Zampieri S, Buratti E, Dominissini S, Montalvo AL, Pittis MG, Bembi B (2011). Splicing mutations in glycogen-storage disease type II: evaluation of the full spectrum of mutations and their relation to patients’ phenotypes. Eur J Hum Genet.

[25] Hermans MM, Kroos MA, de Graaff E, Oostra BA, Reuser AJ (1993). Two mutations affecting the transport and maturation of lysosomal alpha-glucosidase in an adult case of glycogen storage disease type II. Hum Mutat.

[26] Boerkoel CF, Exelbert R, Nicastri C, Nichols RC, Miller FW, Plotz PH (1995). Leaky splicing mutation in the acid maltase gene is associated with delayed onset of glycogenosis type II. Am J Hum Genet.

[27] Huie ML, Chen AS, Tsujino S, Shanske S, DiMauro S, Engel AG (1994). Aberrant splicing in adult onset glycogen storage disease type II (GSDII): molecular identification of an IVS1 (-13T->G) mutation in a majority of patients and a novel IVS10 (+1GT->CT) mutation. Hum Mol Genet.

[28] Goina E, Musco L, Dardis A, Buratti E. Assessment of the functional impact on the pre-mRNA splicing process of 28 nucleotide variants associated with Pompe disease in GAA exon 2 and their recovery using antisense technology. Hum Mutat. 2019;40:2121–30. 10.1002/humu.23867 31301153

[29] Hegde M, Santani A, Mao R, Ferreira-Gonzalez A, Weck KE, Voelkerding KV (2017). Development and validation of clinical whole-exome and whole-genome sequencing for detection of germline variants in inherited disease. Arch Pathol Lab Med.

[30] Byron SA, Van Keuren-Jensen KR, Engelthaler DM, Carpten JD, Craig DW (2016). Translating RNA sequencing into clinical diagnostics: opportunities and challenges. Nat Rev Genet.

[31] Gonorazky HD, Naumenko S, Ramani AK, Nelakuditi V, Mashouri P, Wang P (2019). Expanding the boundaries of RNA sequencing as a diagnostic tool for rare Mendelian disease. Am J Hum Genet.

[32] de Klerk E, t Hoen PA (2015). Alternative mRNA transcription, processing, and translation: insights from RNA sequencing. Trends Genet.

[33] Stein CA, Castanotto D (2017). FDA-approved oligonucleotide therapies in 2017. Mol Ther.

[34] Hoy SM (2018). Patisiran: first global approval. Drugs.

[35] Kuijper EC, Bergsma AJ, Pim Pijnappel WWM, Aartsma-Rus A. Opportunities and challenges for antisense oligonucleotide therapies. J Inherit Metab Dis. 2020. 10.1002/jimd.12251. online ahead of print. 10.1002/jimd.12251 PMC7891411 32391605

[36] Fukuhara Y, Fuji N, Yamazaki N, Hirakiyama A, Kamioka T, Seo JH (2018). A molecular analysis of the GAA gene and clinical spectrum in 38 patients with Pompe disease in Japan. Mol Genet Metab Rep.

