# Supplementary material for: A generic assay for the identification of splicing variants that induce nonsense-mediated decay in Pompe disease
Source: Eur J Hum Genet. 2020 Nov 9;29(3):422–33. doi: 10.1038/s41431-020-00751-3 (PMC7940403; doi:10.1038/s41431-020-00751-3)
Supplement: Supplementary file 1 — Supplementary data [file 41431_2020_751_MOESM1_ESM.docx]

**Supplementary Data**

# Supplementary Text

## Analysis of the c.2481+2T>C GAA variant in Patient 3

Patient 3 was diagnosed with classic infantile onset Pompe disease. Residual GAA enzymatic activity in cultured primary fibroblasts was 1.6 nmol 4-MU/hr/mg protein. Sequence analysis identified compound heterozygous variants causative for the disease. Allele 1 harbored the severe c.1726G>C missense variant, while allele 2 carried the c.2481+2T>C variant that was previously reported as a splicing variant that was not functionally characterized.^1^

To characterize c.2481+2T>C, flanking exon RT-PCR analysis of *GAA* mRNA expression in fibroblasts was performed (Suppl. Figure S5A). An aberrant splicing event was identified in the proximity of *GAA* exon 17: besides the presence of the normal product (K1), a shorter product (K2) was detected at minor amounts. TOPO cloning and subsequent Sanger sequence analysis indicated that product K2 resulted from a complete skip of *GAA* exon 17 (Suppl. Figure S5 and S6A). Skipping of exon 17 led to the loss of 150 bases from the canonical *GAA* mRNA sequence, but did not introduce a frameshift and did not lead to mRNA degradation. Given the relatively small contribution seen on gel, this splicing event could not explain the phenotype of Patient 3 considering that full contribution would show an K1:K2 ratio of 1:1, 1 per allele (Suppl. Figure S5A). Exon internal RT-qPCR indicated an average expression of *GAA* exons of 21.2% compared to the expression in a healthy control suggesting the presence of mRNA decay (Suppl. Figure S5C). To test this, cells were treated with cycloheximide. Flanking exon RT-PCR performed after cycloheximide treatment showed products of aberrant size in proximity of exons 6, 7, 12 and 13, in line with what was found in the healthy control. In addition, novel aberrant products were observed in the proximity of exon 16, 17 and 18 (Suppl. Figure S5D). Product L1 utilized a cryptic splice acceptor site located at c.2332-109 (Suppl. Figure S5D-E and S6B). The retained intronic sequence contained a stop codon that likely induced the activation of the NMD pathway. Utilization of the c.2332-109 splice acceptor site was confirmed after sequencing of the aberrant mRNA species adjacent to exon 17 (Product M1, Suppl. Figure S5D). In addition, product M1 utilized the c.2462 splice donor site 19 bases upstream of the canonical exon 17 donor splice site (Suppl. Figure S5E and S6B). Product M3 and N2 also exploited the cryptic donor splice at c.2462. The subsequent splicing toward the canonical exon 18 acceptor site resulted in a deletion of 19 bases and thus led to a frameshift and likely nonsense mediated decay. Product M4 occurred due to the skipping of exon 17, similar to product K2. Products L2, M2 and N1 were canonically spliced.

*In silico* predictions from four out of five prediction algorithms indicated that the c.2481+2T>C variant caused disruption of the canonical splice donor site of exon 17 (Suppl. Figure S5F). The presence of a cryptic splice donor site at position c.2462 was predicted by three out of five algorithms. Although this cryptic site was correctly predicted, another cryptic donor splice site present at c.2481+23 was also identified. but no aberrant splice products utilizing this splice site were identified, demonstrating the difficulty to predict functional consequences based on *in silico* data. Prediction analysis of the splice site located at c.2332-109 shows a weakly predicted splice acceptor site in three out of five algorithms (Suppl. Figure S6C).

In conclusion, the deleterious effect of the c.2481+2T>C variant was likely caused by a combination of the following: loss of recognition of the canonical exon 17 splice donor site, resulting in a complete skip of exon 17; the utilization of a cryptic splice acceptor site at c.2332-109, and the utilization of a cryptic splice donor site in exon 17. This resulted in the loss of the majority of mRNA products transcribed from this allele.

Analysis of the c.2331+2T>A variant in Patient 4

Patient 4 was diagnosed with Pompe disease at the age of 11. GAA enzymatic activity in primary fibroblasts showed a residual activity of 9.1 nmol 4-MU/hr/mg protein, consistent with a childhood phenotype (Table 1). Genomic DNA analysis identified two compound heterozygous disease-associated variants. Allele 1 harbored the common c.32-13T>G (IVS1) variant that causes aberrant splicing of *GAA* exon 2.^2-7^ Additionally the patient carried the c.510C>T variant on the IVS1 allele, which is known to worsen splicing outcome of the IVS1 variant.^8^ The c.2331+2T>A variant was detected on the second allele. This variant likely affects splicing of *GAA* exon 16 due to its close proximity to the canonical donor splice site of exon 16, however functional data were not available.

Flanking exon RT-PCR analysis on cDNA from primary fibroblasts of Patient 4 indicated the presence of aberrantly spliced products for *GAA* exon 2 (Suppl. Figure S7A, black asterisks). The sizes of these products were consistent with previous reports describing the effect of the IVS1 variant ^3,7^. No additional aberrant splicing events were detected. Next, *GAA* expression levels were determined using exon internal RT-qPCR for all coding exons. The average expression of all *GAA* exons was 8.0% compared to levels in a healthy control (Suppl. Figure S7B). The IVS1 allele commonly generates 5-20% residual canonical *GAA* mRNA expression, suggesting that mRNA transcripts from the c.2331+2T>A allele were partially or completely absent.^8^ To determine whether mRNA degradation occurred as a consequence of the presence of the c.2331+2T>A variant, primary fibroblasts from Patient 4 were treated with cycloheximide. As expected, flanking exon RT-PCR analysis detected aberrant splicing events also seen in healthy controls close to exons 6, 7, 12 and 13 (Suppl. Figure S7C). In addition, aberrant products were observed for exons 16 and 17 (Suppl. Figure S7C, indicated as O1 and P1). Sequencing analysis indicated that these products were generated by utilization of a cryptic splice donor splice site at c.2315 that spliced towards the cryptic splice acceptor site at position c.2332-109 (Suppl. Figure S7D). The resulting products O1 and P1 harbored a frameshift that likely led to mRNA decay (Suppl. Figure S8). Furthermore, the bands at the size of the canonical splice products for exon 16 and 17 (O2 and P2) appeared to be relatively abundant. Sequence analysis of these bands identified additional products (O3 and P3) that utilized the cryptic splice donor site at position c.2315 and the canonical acceptor site of exon 17 at c.2332. These mRNA transcripts lacked the last 16 bases of exon 16, and were subject to degradation due to the introduction of a frameshift (Suppl. Figure S7C-D, products O3 and P3 and Suppl. Figure S8).

*In silico* analysis predicted that recognition of the exon 16 splice donor site is completely lost in the presence of the c.2331+2T>A variant (Suppl. Figure S7E). Moreover, splice prediction algorithms predicted that the cryptic splice sites at c.2315 and c.2332-109 were utilized in the presence of this variant (Suppl. Figure S7E and S6C). This is in agreement with the low residual canonical *GAA* mRNA expression, which likely originates mostly from residual expression from the IVS1 allele. Hence, it is reasonable to assume that expression from the c.2331+2T>A is extremely low or absent.

In conclusion, the c.2331+2T>A variant abrogated canonical splicing of exon 16. Instead, splicing was redirected towards two cryptic splice sites located at c.2315 and c.2332-109. Products that utilized these cryptic sites were out of frame, resulting in mRNA decay.

# Supplementary References

1. Kroos M, Hoogeveen-Westerveld M, Michelakakis H *et al*: Update of the pompe disease mutation database with 60 novel GAA sequence variants and additional studies on the functional effect of 34 previously reported variants. *Hum Mutat* 2012; **33:** 1161-1165.

2. Huie ML, Chen AS, Tsujino S *et al*: Aberrant splicing in adult onset glycogen storage disease type II (GSDII): molecular identification of an IVS1 (-13T-->G) mutation in a majority of patients and a novel IVS10 (+1GT-->CT) mutation. *Hum Mol Genet* 1994; **3:** 2231-2236.

3. Boerkoel CF, Exelbert R, Nicastri C *et al*: Leaky splicing mutation in the acid maltase gene is associated with delayed onset of glycogenosis type II. *Am J Hum Genet* 1995; **56:** 887-897.

4. Dardis A, Zanin I, Zampieri S *et al*: Functional characterization of the common c.-32-13T>G mutation of GAA gene: identification of potential therapeutic agents. *Nucleic Acids Res* 2014; **42:** 1291-1302.

5. Bergsma AJ, Kroos M, Hoogeveen-Westerveld M, Halley D, van der Ploeg AT, Pijnappel WW: Identification and characterization of aberrant GAA pre-mRNA splicing in pompe disease using a generic approach. *Hum Mutat* 2015; **36:** 57-68.

6. van der Wal E, Bergsma AJ, Pijnenburg JM, van der Ploeg AT, Pijnappel W: Antisense Oligonucleotides Promote Exon Inclusion and Correct the Common c.-32-13T>G GAA Splicing Variant in Pompe Disease. *Mol Ther Nucleic Acids* 2017; **7:** 90-100.

7. van der Wal E, Bergsma AJ, van Gestel TJM *et al*: GAA Deficiency in Pompe Disease Is Alleviated by Exon Inclusion in iPSC-Derived Skeletal Muscle Cells. *Mol Ther Nucleic Acids* 2017; **7:** 101-115.

8. Bergsma AJ, In 't Groen SLM, van den Dorpel JJA *et al*: A genetic modifier of symptom onset in Pompe disease. *EBioMedicine* 2019; **43:** 553-561.

9. Li Z, Vuong JK, Zhang M, Stork C, Zheng S: Inhibition of nonsense-mediated RNA decay by ER stress. *RNA* 2017; **23:** 378-394.

Supplementary Figures


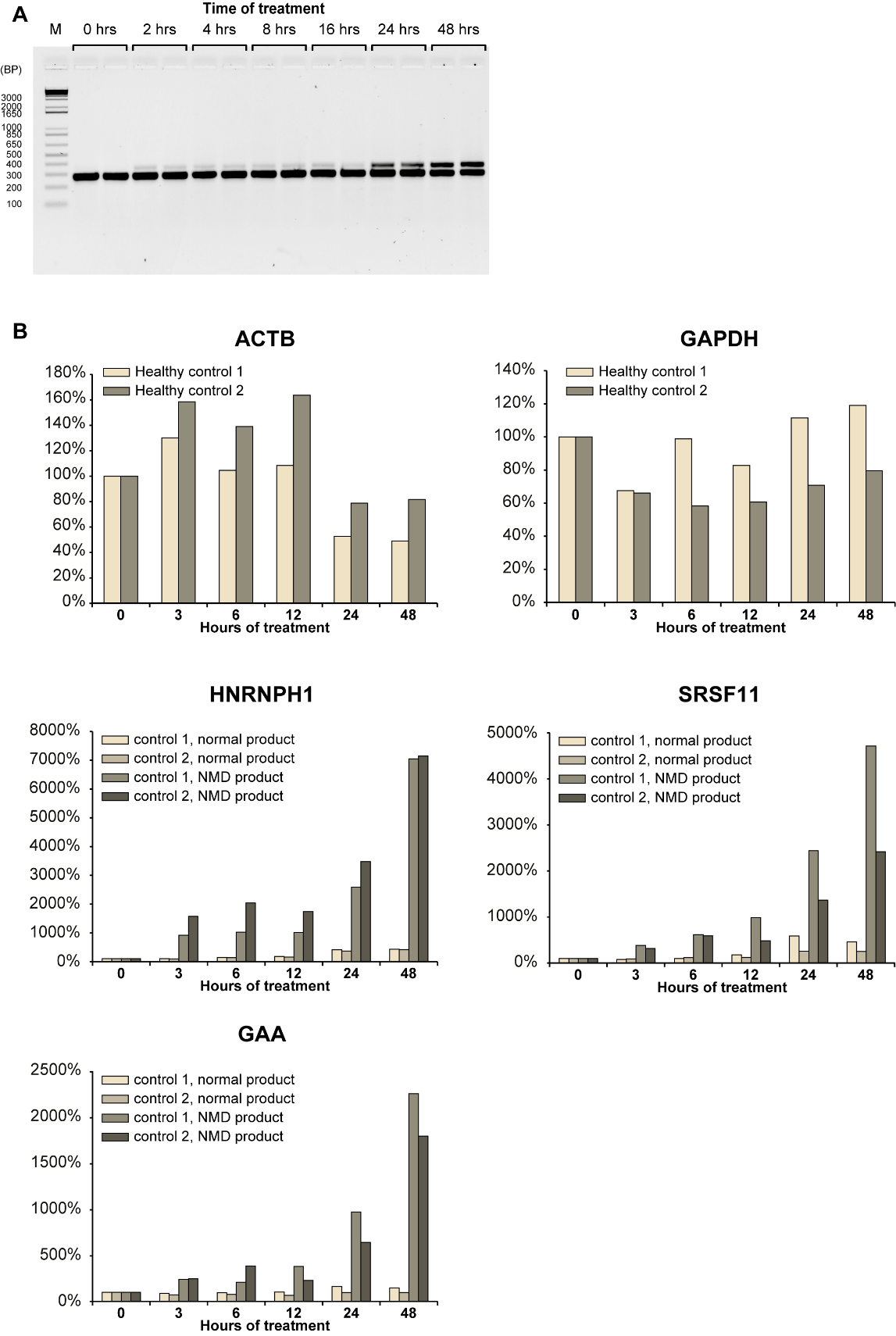


Supplementary Figure S1, optimization of cycloheximide treatment.

A) Flanking exon RT-PCR analysis of *GAA* exon 6. Cells were treated with cycloheximide at a concentration of 100 µg/ml for a specified time period. Analysis revealed that the additional product A (canonical product) was expressed at normal levels, while product B increased in intensity after prolonged cycloheximide treatment. Sequencing analysis of product B revealed that product B was caused by intron 6 retention (Suppl. Figure S2B). B) RT-qPCR analysis of *ACTB* (normalized against *ACTB* 0 hrs treatment) and *GAPDH* (normalized against *ACTB* for each treatment duration). C) RT-qPCR analysis of *HNRNPH1* and *SRSF11*, from which NMD transcripts have been described previously.^9^ Normal transcripts and transcripts that undergo NMD are shown (primers are indicated in Suppl. Table S2). A sharp increase over time is seen in expression of the NMD product compared to the normal product for both genes, indicating that NMD product are not degraded anymore. D) RT-qPCR analysis of exon 1-2 expression (normal *GAA* product) and *GAA* intron 6 expression (NMD product). A 48 hrs treatment regimen was selected for use in further analysis since this treatment showed the most significant change in expression of NMD product.


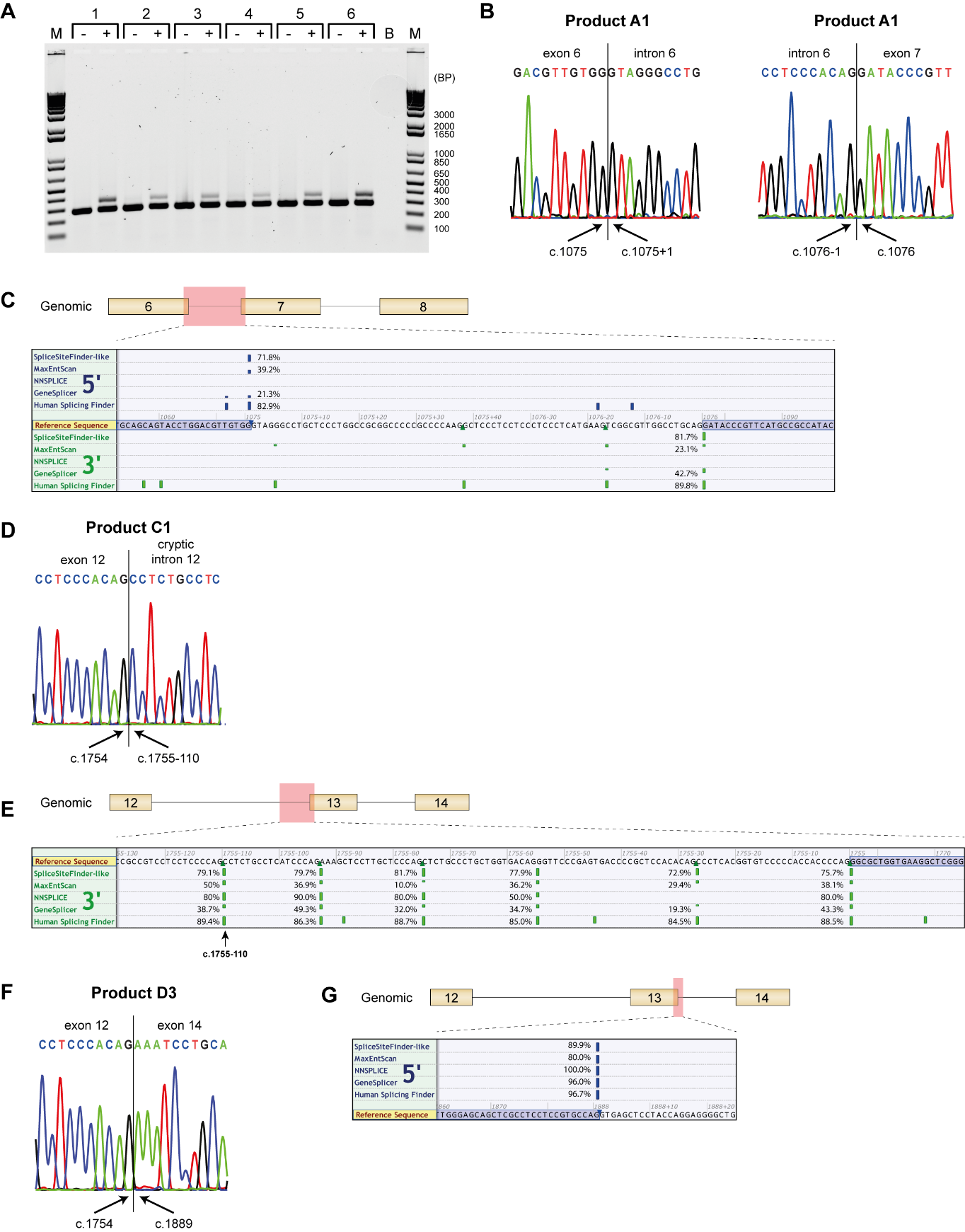


***Supplementary Figure S2, Additional analysis of splicing products in healthy control primary fibroblasts after cycloheximide treatment.***

A) Flanking exon RT-PCR of *GAA* exon 6 in six different healthy control primary fibroblast lines. Number indicates the line. – and + indicate if samples were treated without or with cycloheximide, respectively. B indicates blank sample (water control). Each sample was pooled RT-PCR product from 3 biological replicates. B) Sanger sequencing analysis of products A1 and B1. C) In silico splice prediction of the region containing the 5’ and 3’ splice sites of intron 6 using Alamut®. The pink block in the cartoon highlights the screened region. D) Sanger sequencing analysis of products C1 and D1. E) In silico splice prediction of the region containing the 3’ splice site of intron 12 and upstream region using Alamut®. F) Sanger sequencing analysis of product D3. G) In silico prediction of the 5’ splice site of exon 13 using Alamut®.


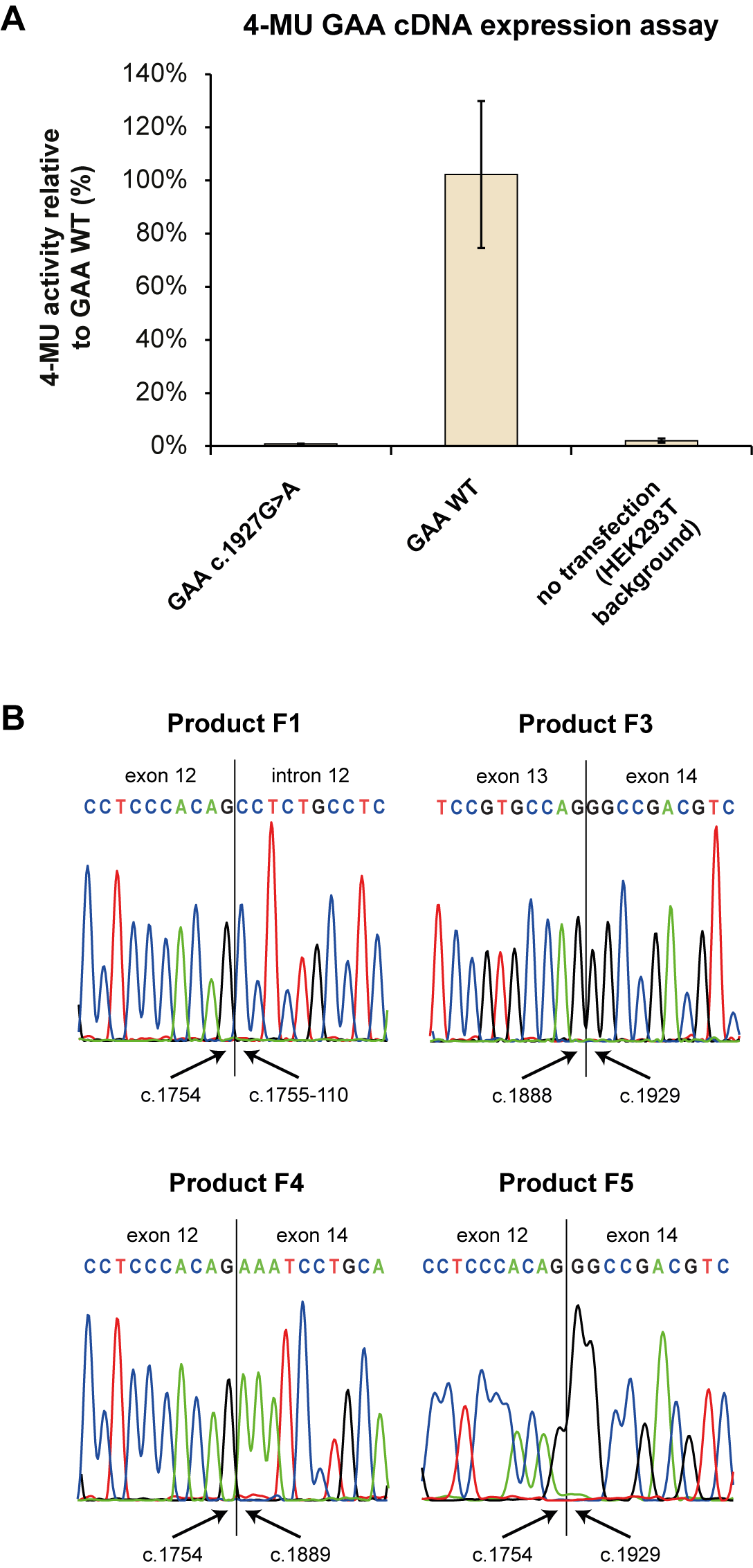


Supplementary Figure S3, Additional analyses performed for Patient 1.

A) Enzymatic activity measurements after transfection of cDNA expression of vectors containing c.1927G>A *GAA* cDNA and WT *GAA* cDNA in HEK293T cells. B) Sequencing data at splice junctions present in products F1-F5 from Figure 2 are shown.


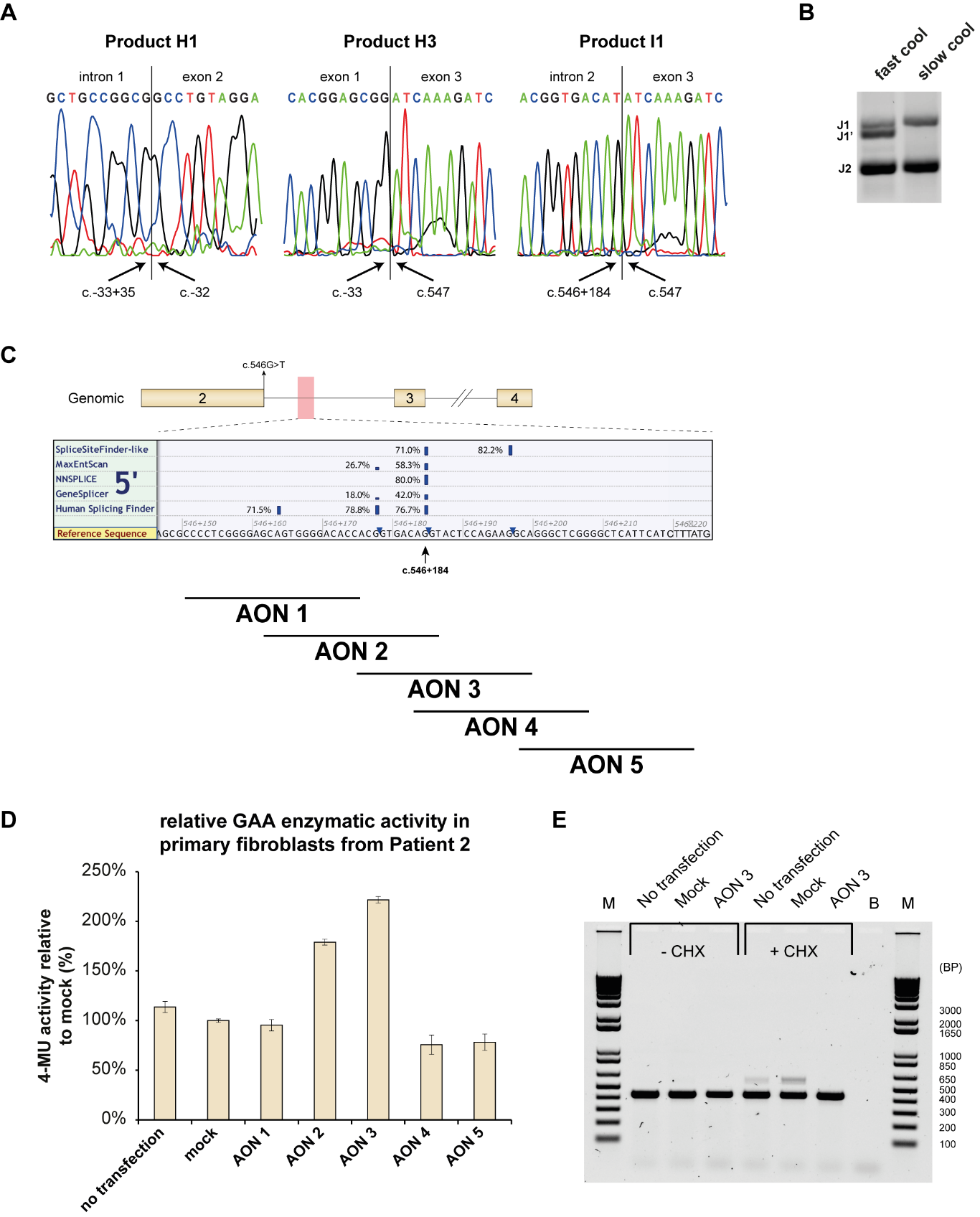


Supplementary Figure S4, Optimization of treatment with AONs in primary fibroblasts from Patient 2.

A) Sequencing analysis of splice junctions in products H1, H3 and I1. B) Analysis of the products J1 and J1’ from Figure 3 Product J1’ is visible when the PCR sample is cooled down quickly, but disappears when the sample is slowly cooled down, indicating secondary structure formation. C) *In silico* splice predictions of the region containing the natural cryptic splice site in *GAA* intron 2 utilized in product I1 using Alamut®. In addition, the target region of five AONs that were designed to block utilization of the cryptic splice site are indicated D) AON treatment of primary fibroblasts from Patient 2, grown in the absence of cycloheximide. Five AONs were tested (targets indicated in (C), sequences indicated in Supp. Table S3). Data are shown as mean +/- S.D. from three biological replicates. E) Flanking exon RT-PCR analysis of *GAA* exon 3 in Patient 2 primary fibroblasts treated with AON 3 and cycloheximide. – CHX and + CHX indicate if samples were treated without or with cycloheximide, respectively. B indicates blank sample (water control). Each sample was pooled RT-PCR product from 3 biological replicates. We note that quantification of mRNA expression from the c.546+184 is obscured by the c.1798C>G allele, which is expressed at the mRNA level but results in enzymatically inactive GAA protein.


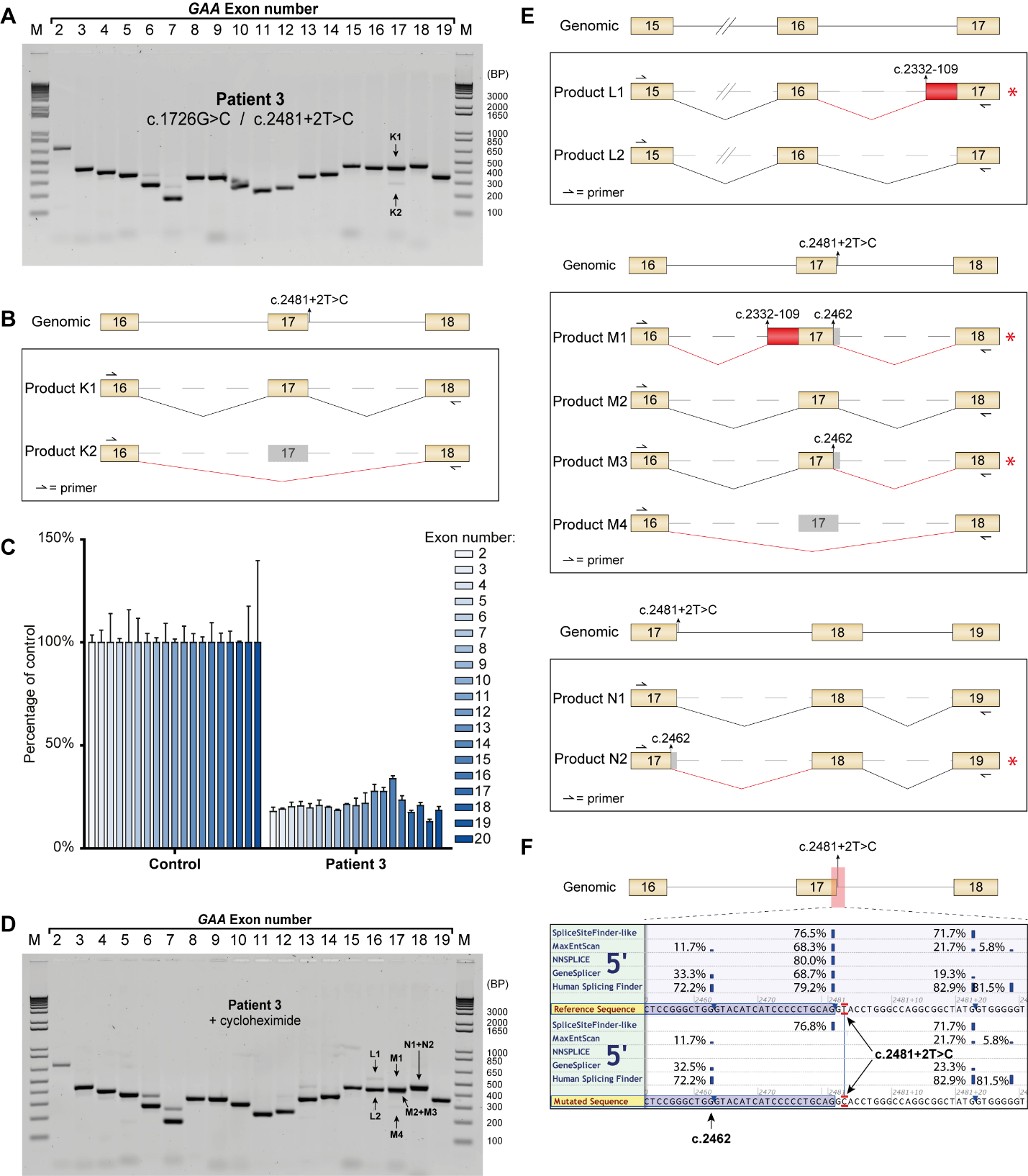


Supplementary Figure S5, Analysis of aberrant splicing products in Patient 3.

A) Flanking exon RT-PCR of patient 3 primary fibroblasts. B) Cartoon of products identified after TOPO cloning and sequencing of specific products from the flanking exon RT-PCR of exon 17. Product numbers refer to products highlighted in A. Red lines indicate aberrant splicing events. Grey area’s indicate skipping of canonical *GAA* mRNA sequence. C) Exon-internal RT-qPCR for all coding exons of patient 3 compared to healthy control. Data are shown as mean +/- S.D. from three technical replicates. D) Same as in A, but after cycloheximide treatment. E) Cartoons of products present in PCR samples for exons 16, 17 and 18. Product numbers refer to product highlighted in D. Red asterisks indicate products undergoing NMD. Red boxes are intronic sequences present in the *GAA* mRNA transcripts. F) Output of splice prediction performed in Alamut®. Five splice site prediction algorithms are shown for the area indicated in red, with or without the presence of the c.2481+2T>C variant. The location of the cryptic donor site used in products L1, L3 and M2 is highlighted.


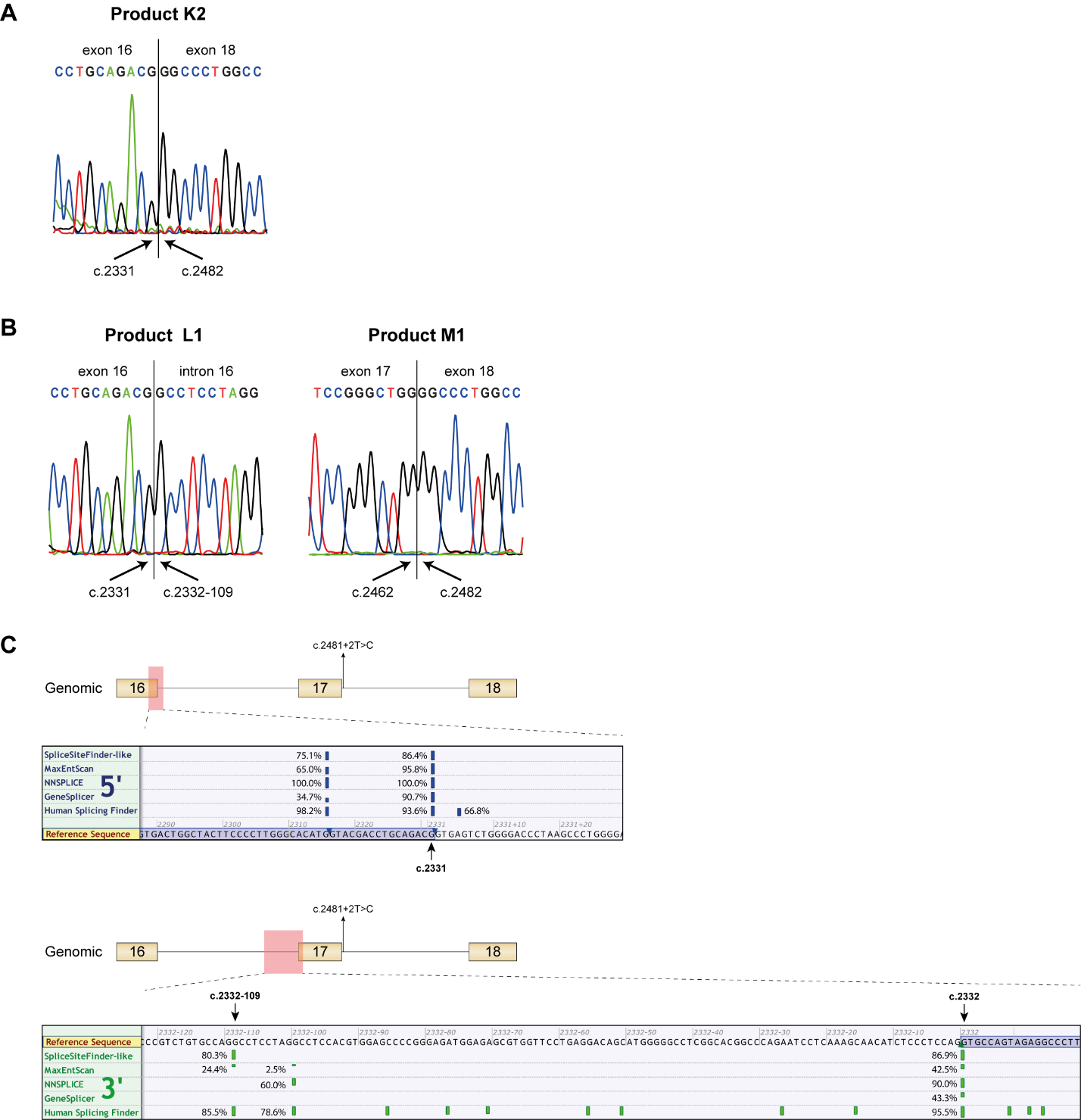


Supplementary Figure S6, In silico and sequencing analysis of splicing in primary fibroblasts derived from Patient 3 after cycloheximide treatment.

A) Sanger sequencing results of splice junctions present in products K1, L1, M1, M3, M4 and N2 from Supplementary Figure S4. B) *In silico* splice prediction of the region from the cryptic splice site utilized by products L1 and M1 to the canonical splice site of exon 17.


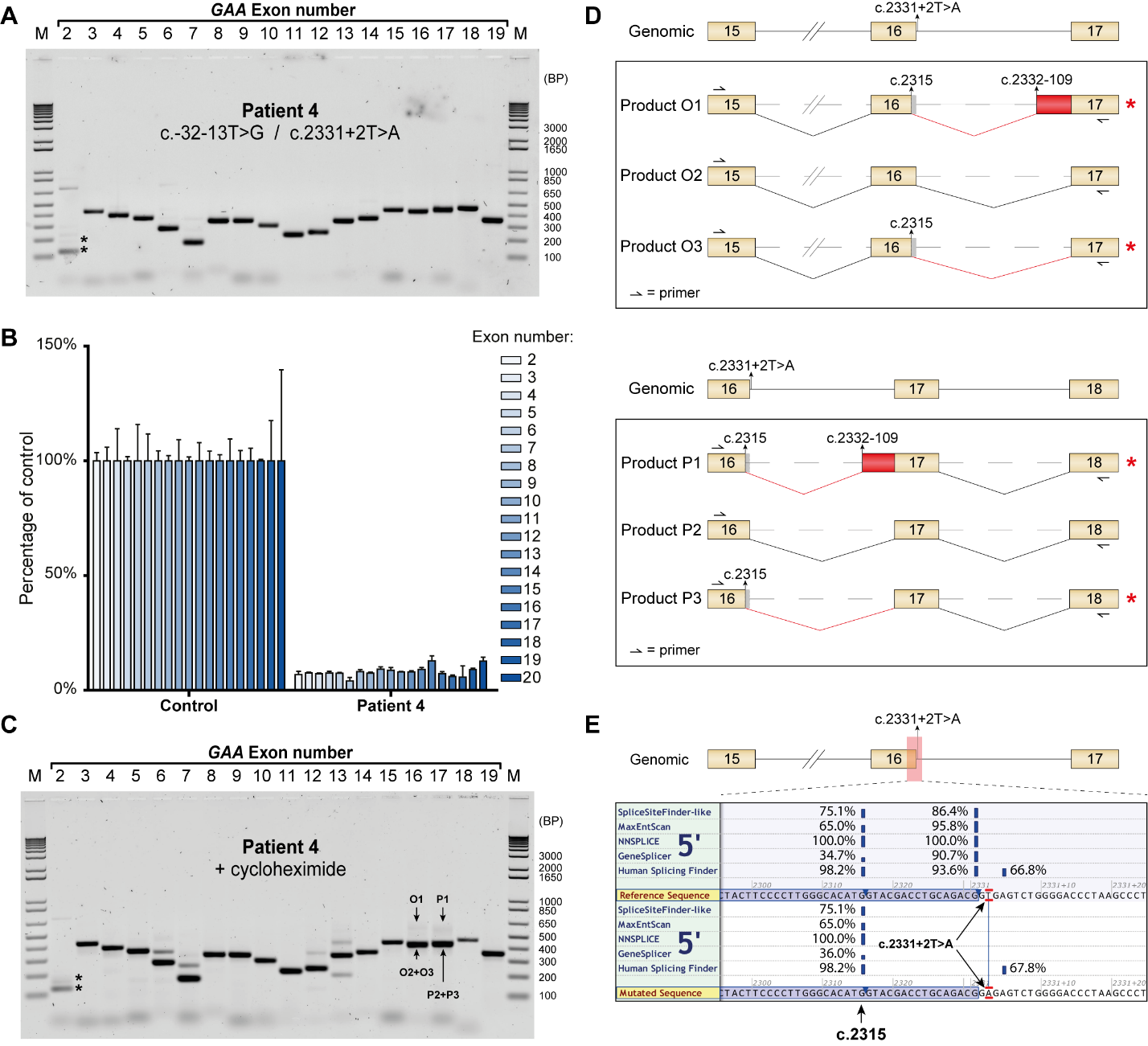


Supplementary Figure S7, Analysis of aberrant splicing products in Patient 4.

A) Flanking exon RT-PCR of Patient 4 primary fibroblasts. Black asterisks indicate aberrant products caused by the c.32-13T>G variant. B) exon-internal RT-qPCR for all coding exons of Patient 4 compared to healthy control. Data are shown as mean +/- S.D. from three technical replicates. C) Same as in A, but after cycloheximide treatment. D) Cartoons of products present in PCR samples for exons 16 and 17. Product numbers refer to product highlighted in C. Red asterisks indicate products undergoing NMD. Red boxes indicate non-canonical sequences present in the *GAA* mRNA transcripts. Grey area’s highlight skipping of canonical *GAA* mRNA sequence. E) Output of splice prediction performed in Alamut®. Five splice site prediction algorithms are shown for the area indicated in red, with or without the presence of the c.2331+2T>A variant. The location of the cryptic donor site utilized in products O1, O3, P1 and P3 is highlighted.


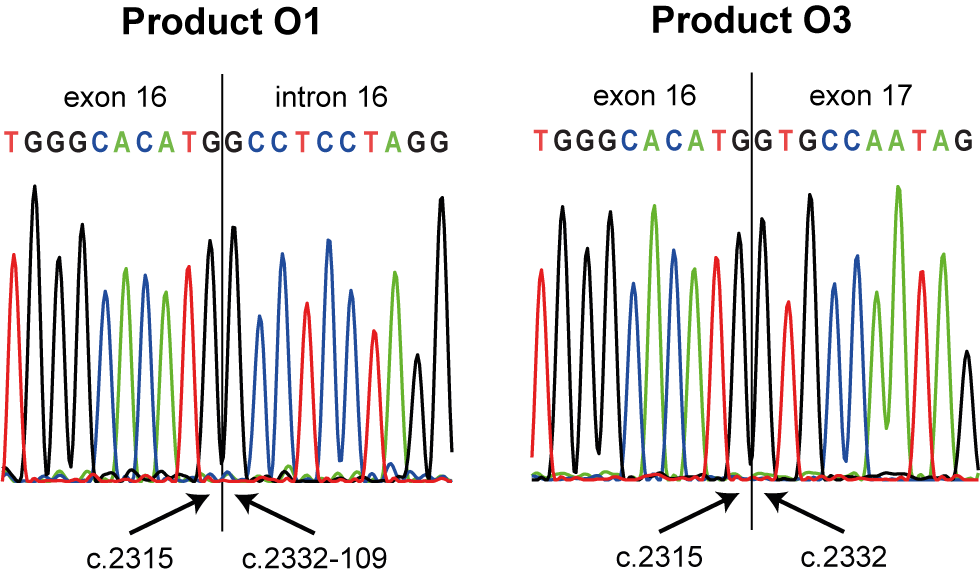


Supplementary Figure S8, sequence analysis additional product identified in Patient 4.

Sequencing data at splice junctions present in products O1, O3, P1 and P3 from Supplementary Figure S6 are shown.

**Supplementary Table S1, Sequences of antisense oligonucleotides used in this study**

| AON nr. | AON target | AON sequence |
| --- | --- | --- |
| 1 | *GAA* c.546+151_+175 | TGGTGTCCCCACTGCTCCCCGAGGG |
| 2 | *GAA* c.546+162_+186 | ACCTGTCACCGTGGTGTCCCCACTG |
| 3 | *GAA* c.546+175_+199 | TGCCTTCTGGAGTACCTGTCACCGT |
| 4 | *GAA* c.546+184_+208 | CCCGAGCCCTGCCTTCTGGAGTACC |
| 5 | *GAA* c.546+199_+223 | TAAAGATGAATGAGCCCCGAGCCCT |

**Supplementary Table S2, Flanking exon RT-PCR primers used in this study.**

| Amplified exon: | Forward^1^: | Reverse^2^: | Full length product: | Size amplified exon: | Skipped product: |
| --- | --- | --- | --- | --- | --- |
| *GAA* Exon 2 | AAACTGAGGCACGGAGCG | GAAGGGCTCCTCGGAGAA | 705 | 578 | 127 |
| *GAA* Exon 3 | AGCTCCTCTGAAATGGGCTACA | GCAAGGTCCCGGTTCCACA | 428 | 146 | 282 |
| *GAA* Exon 4 | GCTAACAGGCGCTACGAGGT | TGCTGTTTAGCAGGAACACCC | 388 | 166 | 222 |
| *GAA* Exon 5 | CTGTTCTTTGCGGACCAGTTC | CCACAACGTCCAGGTACTGCT | 361 | 97 | 265 |
| *GAA* Exon 6 | GGTCTCACCCTTTCTACCTGG | GTGATAGCGGTGGAGGAGTAG | 274 | 120 | 154 |
| *GAA* Exon 7 | CAGCAGTACCTGGACGTTGTG | AGTCCATGTAGTCCAGGTCGTT | 175 | 119 | 56 |
| *GAA* Exon 8 | CGTTCATGCCGCCATACT | GGTCTCGTTGGTGATGAAAAC | 335 | 132 | 203 |
| *GAA* Exon 9 | GACGTCCAGTGGAACGACCT | ACCTGGTCATGGAACTCAGC | 335 | 111 | 224 |
| *GAA* Exon 10 | GATCCTGCCATCAGCAGCT | TGGGTTCTCCAGCTCATTGT | 297 | 114 | 183 |
| *GAA* Exon 11 | AGGACATGGTGGCTGAGTTC | CGTAGAGGTTGTGCAGGTTGTA | 228 | 85 | 143 |
| *GAA* Exon 12 | AACGAGCCTTCCAACTTCATC | GAGCGGGAGATCACAAATGG | 245 | 118 | 127 |
| *GAA* Exon 13 | CACCAGTTTCTCTCCACACACTA | GTTCCGCATGAAGGGGTAGA | 337 | 134 | 202 |
| *GAA* Exon 14 | ACACGCCCATTTGTGATCTC | GTGTAGAGGTGGGGGAGGAGT | 356 | 152 | 204 |
| *GAA* Exon 15 | AAATCCTGCAGTTTAACCTGCTG | GCAGGTCGTACCATGTGCC | 438 | 149 | 289 |
| *GAA* Exon 16 | GAGCCGTACAGCTTCAGCGA | ATGTACCCAGCCCGGAGGT | 422 | 142 | 280 |
| *GAA* Exon 17 | CCTGGACTGTGGACCACCA | CAGGAAGATGACCTGTGTGTAGG | 428 | 150 | 278 |
| *GAA* Exon 18 | GTGCCAGTAGAGGCCCTTG | GGCTGTAGGTGAAGTTGGAGAC | 457 | 165 | 292 |
| *GAA* Exon 19 | TCACAACCACAGAGTCCCG | AGAAACTGCTCTCCCATCAACA | 352 | 153 | 199 |

^1^ The forward primer anneals to the exon upstream of the amplified exon.

^2^ The reverse primer anneals to the exon downstream of the amplified exon.

**Supplementary Table S3, RT-qPCR primers used in this study**

| Target | Forward | Reverse | Product size (bp) |
| --- | --- | --- | --- |
| *ACTB* | AACCGCGAGAAGATGACCC | GCCAGAGGCGTACAGGGATAG | 96 |
| *GAA* Exon 2 | AGCTCCTCTGAAATGGGCTACAC | GGTTCTCAGTCTCCATCATCACG | 109 |
| *GAA* Exon 3 | ATCCAGCTAACAGGCGCTAC | GCTCCTCGGAGAACTCCAC | 96 |
| *GAA* Exon 4 | CTGTTCTTTGCGGACCAGTT | CTGAGCATCAGGGGACTGAG | 95 |
| *GAA* Exon 5 | CGAACCTCTACGGGTCTCAC | TGCTGTTTAGCAGGAACACC | 81 |
| *GAA* Exon 6 | CTTAGCTGGAGGTCGACAGG | CACAACGTCCAGGTACTGCT | 93 |
| *GAA* Exon 7 | CGTTCATGCCGCCATACT | GGTCATGTTCTCCACCACCT | 95 |
| *GAA* Exon 8 | GACGTCCAGTGGAACGACCT | GAAGTCCCGGAAGCCATC | 78 |
| *GAA* Exon 9 | ATCCTGCCATCAGCAGCTC | GGTCTCGTTGGTGATGAAAA | 89 |
| *GAA* Exon 10 | CACTGCCTTCCCCGACTT | ACCTGGTCATGGAACTCAGC | 78 |
| *GAA* Exon 11 | ACATGAACGAGCCTTCCAAC | ACGTAGGGTGGGTTCTCCAG | 79 |
| *GAA* Exon 12 | CCTCCAGCCACCAGTTTCTCT | TGTGGGAGGCGATGGCTT | 78 |
| *GAA* Exon 13 | GACACGCCCATTTGTGATCT | CCAGGAGCTCCACACGTC | 88 |
| *GAA* Exon 14 | CTCAGAGGAGCTGTGTGTGC | CAGACTGAGCAGGCTGTTGT | 82 |
| *GAA* Exon 15 | CAGCAGGCCATGAGGAAG | GGCCTGGTGGAACAGTGTG | 75 |
| *GAA* Exon 16 | CCCAAGGACTCTAGCACCTG | CAAGGGGAAGTAGCCAGTCA | 114 |
| *GAA* Exon 17 | GTGCCAGTAGAGGCCCTTG | GAGGTGGACGTTGATGGTGT | 123 |
| *GAA* Exon 18 | GCCTCACAACCACAGAGTCC | TCTCTCCATCGTCCCAGAAC | 102 |
| *GAA* Exon 19 | TGCAGAAGGTGACTGTCCTG | GGGCTGTAGGTGAAGTTGGA | 88 |
| *GAA* Exon 20 | GGGCGGAGTGTGTTAGTCTC | CTCCAGGTGACACATGCAAC | 110 |
| *GAPDH* | CGGTTTCTATAAATTGAGCCCG | GCGACGCAAAAGAAGATGC | 86 |
| *HNRNPH1* | GGTCGCAGTGTATGTTTGATG | GGCGTTTGTTGGGGTTACT | 92 |
| *HNRNPH1 NMD* | CAACCTCAGTGGACAAGGTG | CCTCATATTCTGCGGGATGA | 100 |
| *SRSF11* | TCCAGACTCAGCAGTTGTGG | GGCTGAACCAGGGAAAAGA | 100 |
| *SRSF11 NMD* | TCCAGACTCAGCAGTTGTGG | TCTCATCAGGAATAACTCTTCAGC | 102 |
| *GAA* Intron 6 | CCTCATGAAGTCGGCGTTG | CCTGGTCATGTTCTCCACCA | 131 |
| *GAA* Intron 2 | GGAGGTGTGAGCAGACAATG | GTAGCGCCTGTTAGCTGGAT | 98 |
| *Neomycin* | TCATCTCACCTTGCTCCTGC | GTGGTCGAATGGGCAGGTAG | 96 |

**Supplementary Table S4, prediction of splice site strength of exon 2 splice donor and cryptic intron 2 splice donor.**

| Prediction algorithm | *GAA* c.546G exon 2 splice donor site | *GAA* c.546T exon 2 splice donor site | *GAA* c.546+184 intron 2 cryptic splice donor site |
| --- | --- | --- | --- |
| *SpliceSiteFinder-like* | 70.6% | 0.0% | 71.0% |
| *MaxEntScan* | 56.7% | 0.0% | 58.3% |
| *NNSPLICE* | 40.0% | 0.0% | 80.0% |
| *GeneSplicer* | 67.3% | 0.0% | 42.0% |
| *Human Splicing Finder* | 82.6% | 71.8% | 76.7% |
